# Gut inflammation is associated with structural spinal damage in axial spondyloarthritis – results from the observational SPARTAKUS cohort

**DOI:** 10.1186/s13075-025-03663-z

**Published:** 2025-10-21

**Authors:** Johan Karlsson Wallman, Elisabeth Mogard, Jonas Sagard, Kristofer Andréasson, Jan Marsal, Fatih Inci, Mats Geijer, Tor Olofsson, Elisabet Lindqvist

**Affiliations:** 1 Department of Clinical Sciences Lund, Rheumatology, Lund University, Lund, Sweden; 2 Department of Rheumatology, Skåne University Hospital, Region Skåne, Lund, Sweden; 3 Department of Clinical Sciences Lund, Gastroenterology, Lund University, Lund, Sweden; 4 Department of Gastroenterology, Skåne University Hospital, Region Skåne, Lund/Malmö, Sweden; 5 Department of Radiology, Institute of Clinical Sciences, Sahlgrenska Academy, University of Gothenburg, Gothenburg, Sweden; 6 Department of Radiology, Sahlgrenska University Hospital, Region Västra Götaland, Gothenburg, Sweden; 7 Department of Clinical Sciences, Radiology, Lund University, Lund, Sweden

**Keywords:** Spondyloarthritis, Non-radiographic axial spondyloarthritis, Ankylosing spondylitis, Inflammatory bowel disease, Gut inflammation, Calprotectin, Structural damage, Radiographic progression

## Abstract

**Background:**

In axial spondyloarthritis (axSpA), 5–10% of patients have comorbid inflammatory bowel disease (IBD). Beyond that, 50–60% display histologic inflammation in ileum/colon biopsies, and fecal calprotectin (F-calprotectin) is elevated in relation to healthy controls. Prior studies have shown such, often subclinical, gut inflammation in axSpA to be associated with more active disease, as measured by clinical indices as well as magnetic resonance imaging – both known risk factors for structural spinal damage development. In light of this, in the current study we aimed to examine whether gut inflammation, assessed by F-calprotectin, is associated with more structural spinal damage in axSpA.

**Methods:**

Patients with well-characterized non-radiographic or radiographic axSpA (nr-axSpA/r-axSpA; *n* = 76/152), according to ASAS or modified New York criteria, enrolled in a population-based cohort study in southern Sweden, were assessed for structural spinal damage (modified Stoke ankylosing spondylitis spinal score [mSASSS]) and gut inflammation (F-calprotectin). mSASSS values were compared between patients with normal (< 50 mg/kg), moderately elevated (50–149 mg/kg) or distinctly elevated (≥ 150 mg/kg) F-calprotectin, reflecting no/some/evident gut inflammation, respectively (one-way ANOVA). Moreover, logistic regression was applied to explore if elevated F-calprotectin (≥ 50 mg/kg) was associated with mSASSS values above the median, adjusted for sex, symptom duration, HLA-B27 status, smoking, CRP, NSAID and anti-TNF therapy. Analyses limited to r-axSpA were also performed.

**Results:**

In both axSpA patients overall and separately in r-axSpA, mSASSS distributions differed significantly between subjects with normal/moderately/distinctly elevated F-calprotectin, with more damage observed in those with higher F-calprotectin levels. Furthermore, elevated F-calprotectin (≥ 50 mg/kg) was associated with mSASSS values above the median, in both the entire axSpA group (adjusted odds ratio [OR] 2.2 [95%CI 1.1–4.2]); and in r-axSpA alone (adjusted OR 2.9 [1.2–7.1]).

**Conclusion:**

In the current study, the presence of gut inflammation, assessed by F-calprotectin, was cross-sectionally associated with more structural damage in the spine in patients with axSpA, even after adjustments for known risk factors for spinal damage. Prospective studies are, however, needed to investigate whether gut inflammation may be a predictor of spinal radiographic progression in axSpA.

**Supplementary Information:**

The online version contains supplementary material available at 10.1186/s13075-025-03663-z.

## Background

Axial spondyloarthritis (axSpA) is characterized by inflammation of the sacroiliac (SI) joints and spine, which over time may cause structural damage and functional impairment. Both clinically and genetically, axSpA is closely associated with inflammatory bowel disease (IBD), and 5–10% of axSpA patients have comorbid IBD. Beyond this, 50–60% of SpA patients display histologic inflammation in ileum and/or colon biopsies, most of whom lack typical gastrointestinal IBD symptoms [1–3]. Such subclinical gut inflammation in axSpA is also reflected in studies of fecal (F) calprotectin (a standard, clinical disease activity biomarker in IBD [4]), which is elevated in axSpA versus healthy controls [5, 6].

Since structural SI-joint/spinal damage accumulation is a central long-term outcome in axSpA [7], characteristics signaling an increased risk for such disease progression are of important prognostic value. Known risk factors include male sex, HLA-B27 positivity, smoking and more active disease, defined by worse clinical indices, more bone marrow edema (BME) on magnetic resonance imaging (MRI) and elevated C-reactive protein (CRP) [8–11]. Interestingly, several studies indicate a link between gut inflammation (defined by mucosal histology or elevated F-calprotectin) and more active disease in axSpA [2, 5, 6, 12–15]. Yet, it remains unknown whether this means that gut inflammation is also a risk factor for structural spinal damage progression.

In light of this, we aimed to assess whether gut inflammation, measured by elevated F-calprotectin, is cross-sectionally associated with more structural spinal damage in axSpA.

## Methods

### Study population

Patients from the population-based SPARTAKUS cohort of well-characterized axSpA cases were assessed. The inclusion/classification for this cohort is described elsewhere [5]. In brief, all individuals from a defined area of Skåne County, Sweden, with ≥ 1 outpatient visit to the Department of Rheumatology, Skåne University Hospital, 2011–2014 with an ICD-10 diagnosis consistent with axSpA (M45.9/M46.0/M46.1/M46.8/M46.9), were invited to enroll. Subjects with undifferentiated SpA diagnoses (M46.8/M46.9) had to report back pain ≥ 3 months with onset before age 45 years to be eligible. At enrolment (2015–2019), subjects were thoroughly examined, including questionnaires, clinical examinations, blood/feces/urine sampling and imaging. The latter comprised radiographs and, if needed for classification, also MRI of the SI-joints, as well as radiographs of the lumbar and cervical spine to assess structural damage by the modified Stoke ankylosing spondylitis spinal score (mSASSS). All imaging was scored by the same experienced musculoskeletal radiologist (MG). To assess reliability of the mSASSS scores, 40 randomly selected cases were, however, also read by a second experienced musculoskeletal radiologist (FI), and inter-reader agreement analyzed. Of the 344 individuals enrolled in SPARTAKUS, 266 fulfilled ASAS (Assessment of SpondyloArthritis international Society) axSpA and/or modified New York (mNY) classification criteria (*n* = 86/180 for non-radiographic/radiographic axSpA [nr-axSpA/r-axSpA], encompassing eight r-axSpA patients fulfilling only mNY criteria). Of these, 228 (n[nr-axSpA/r-axSpA] = 76/152) had available information on both mSASSS (missing *n* = 10) and F-calprotectin (see below; missing *n* = 30) and comprised the current study population.

### Outcomes

Structural spinal damage was assessed by mSASSS (range 0–72) and gut inflammation by F-calprotectin (ELISA; Calpro AS, Oslo). Regarding F-calprotectin, values < 50 mg/kg are considered normal by the manufacturer [4, 16], whereas moderately elevated values up to 150 mg/kg (reflecting some degree of gut inflammation) have been reported as borderline for the detection of IBD, where repeated F-calprotectin testing could be considered before deciding on endoscopic examination [4, 17].

To assess if presence of gut inflammation is cross-sectionally associated with more structural spinal damage in axSpA, mSASSS of patients with normal F-calprotectin (< 50 mg/kg) were contrasted to those with moderately elevated (50–149 mg/kg; reflecting some degree of gut inflammation) or distinctly elevated (≥ 150 mg/kg; reflecting evident gut inflammation) values. Furthermore, we examined whether presence of gut inflammation, determined as (I) F-calprotectin ≥ 50 mg/kg (yes/no); and (II) higher F-calprotectin, as a continuous measure, was associated with an mSASSS above the median of all assessed patients, while adjusting for potential confounders known to influence the risk for structural damage accumulation: sex, symptom duration, HLA-B27 status, smoking (ever/never), CRP (continuous measure), non-steroidal anti-inflammatory drug (NSAID) use (as ASAS 3-month NSAID-score) and anti-TNF (tumor necrosis factor) therapy use (yes/no) at the time of examination [9].

Apart from assessing the entire axSpA group (nr-axSpA and r-axSpA), analyses were also performed separately for r-axSpA patients, since both mSASSS and F-calprotectin were expected to be higher in r-axSpA [5]. Analyses were also repeated after exclusion of patients with IBD (n[nr-axSpA/r-axSpA] = 3/19) as a sensitivity analysis. Moreover, since dietary habits and certain gastrointestinal comorbidities other than IBD may potentially affect F-calprotectin values, additional adjustment for these factors was explored in a further sensitivity analysis (for more details, see Additional file 1).

### Statistics

Comparison of mSASSS between patients with various F-calprotectin levels (< 50/50–149/≥150 mg/kg) was performed by one-way analysis of variance (ANOVA), using Log_10_-transformed mSASSS as outcome due to its skewed distribution. Logistic regression, crude and adjusted for the covariates detailed above, was applied to assess whether presence of gut inflammation was associated with an mSASSS above the median. For this purpose, F-calprotectin was examined both as a categorical measure (≥ 50 mg/kg, reflecting at least some gut inflammation) and as a continuous variable (Log_10_-transformed F-calprotectin, due to skewness). When used for adjustment, CRP and ASAS 3-month NSAID-scores were also Log_10_-transformed due to skewness.

Inter-reader agreement in mSASSS was evaluated with an intraclass correlation coefficient (ICC) two-way mixed-effect model, with single measurement and absolute agreement. ICC values < 0.50/0.50–0.75/0.75–0.90/>0.90 indicate poor/moderate/good/excellent agreement [18]. To examine whether patients not included due to lack of mSASSS and/or F-calprotectin data differed from those included, patient characteristics were compared by Chi^2^ or Mann Whitney U-test.

## Results

### Patient characteristics

Included patients (53% male subjects) had overall well-established axSpA with a mean (SD) symptom duration of 25 (14) years. Similarities/differences between the nr-axSpA and r-axSpA subsets were in accordance with the literature (Table 1). F-calprotectin was elevated (≥ 50 mg/kg) in 1/3 of patients, with higher values in r-axSpA than nr-axSpA, and 22 subjects (9.6%) had comorbid IBD (of whom 86% were r-axSpA). The median (interquartile range) mSASSS in the entire study population was 2.0 (0 to 8.0), with median values of 0 and 5.0 for nr-axSpA and r-axSpA, respectively. NSAIDs/anti-TNF therapy were used in 61%/42% of all patients.

The observed ICC for mSASSS was 0.895 (95%CI 0.810–0.943), indicating a good to excellent inter-reader agreement. Characteristics of the patients excluded due to missing mSASSS and/or F-calprotectin data (*n* = 38) were similar to those included, except for a higher patient´s VAS (visual analogue scale) global and somewhat worse dietary habits (Additional file 1, Supplementary Table S1).

**Table 1:** Characteristics of the study population

	All axSpA	Non-radiographic axSpA	Radiographic axSpA
	n = 228	n = 76	n = 152
**Male sex**, n (%)	121 (53%)	29 (38%)	92 (61%)
**Age**, years	51 (13)	46 (12)	53 (13)
**Smoking ever**, n (%)	81 (36%)	18 (24%)	63 (42%)
**Dietary index** ^a^, 0–12 points	7.2 (2.1)	7.5 (2.3)	7.0 (2.0)
**Family history of SpA**, n (%)	97 (43%)	32 (42%)	65 (43%)
**Symptom duration**, years	25 (14)	20 (11)	28 (14)
**HLA-B27 positive**, n (%)	198 (87%)	69 (91%)	129 (85%)
**Back pain ≥ 3 months**:			
With onset < 45 years, n (%)	220 (98%)	76 (100%)	144 (97%)
Improved by exercise and not relieved by rest, n (%)	169 (76%)	59 (78%)	110 (75%)
**Inflammatory back pain (ASAS definition)**, n (%)	196 (86%)	64 (84%)	132 (87%)
**Sagittal lumbar flexion (Modified Schober´s test)**, cm	4.3 (2.9)	4.5 (1.1)	4.2 (3.5)
**Lateral lumbar flexion** ^b^, cm	13.9 (5.2)	15.8 (4.7)	12.9 (5.3)
**Chest expansion**, cm	5.0 (1.3)	4.8 (1.8)	5.4 (1.9)
**Sacroiliitis on plain X-ray**, n (%)	152 (67%)	0 (0%)	152 (100%)
**mSASSS**			
Mean (SD)	9.3 (17)	1.8 (5.4)	13 (20)
Median (IQR)	2.0 (0.0–8.0)	0.0 (0.0–2.0)	5.0 (0.0–18)
**SI joint MRI available**, n (%)	124 (54%)	49 (64%)	75 (49%)
SI joint bone marrow edema on MRI ^c^, n (%)	65 (52%)	22 (45%)	43 (57%)
**Good response of back pain to NSAIDs**, n (%)	176 (77%)	58 (76%)	118 (78%)
**Elevated CRP in the presence of back pain**, n (%)	139 (61%)	40 (53%)	99 (65%)
**Peripheral arthritis**, n (%)	118 (52%)	45 (59%)	73 (48%)
**Dactylitis**, n (%)	24 (11%)	13 (17%)	11 (7.2%)
**Heel enthesitis**, n (%)	101 (44%)	39 (51%)	62 (41%)
**History of uveitis**, n (%)	95 (42%)	25 (33%)	70 (46%)
**Skin and/or nail psoriasis**, n (%)	20 (8.8%)	6 (7.9%)	14 (9.2%)
**Inflammatory bowel disease**, n (%)	22 (9.6%)	3 (3.9%)	19 (13%)
**Other gastrointestinal comorbidity** ^d^, n (%)	36 (17%)	6 (8.3%)	30 (21%)
**ASDAS**	1.8 (0.9)	1.7 (0.9)	1.8 (0.9)
**BASDAI**	3.0 (2.2)	2.9 (2.0)	3.0 (2.2)
**BASFI**	2.0 (2.1)	1.8 (1.8)	2.1 (2.3)
**BASMI**	3.0 (1.6)	2.3 (1.1)	3.3 (1.7)
**VAS pain**, mm	30 (25)	29 (21)	31 (26)
**VAS global**, mm	30 (24)	30 (22)	30 (26)
**CRP**, mg/L	3.4 (4.9)	2.4 (2.8)	3.9 (5.7)
**F-calprotectin**, mg/kg			
Mean (SD)	66 (113)	40 (49)	80 (132)
Median (IQR)	30 (14–65)	25 (11–45)	32 (14–84)
**Elevated F-calprotectin ≥ 50 mg/kg**, n (%)	76 (33%)	18 (24%)	58 (38%)
**ASAS 3-month NSAID score**	31 (41)	29 (41)	32 (42)
**Ongoing csDMARD**	48 (21%) ^e^	15 (20%)	33 (22%) ^e^
Methotrexate, n (%)	29 (13%)	8 (11%)	21 (14%)
Sulfasalazine, n (%)	14 (6.1%)	5 (6.6%)	9 (5.9%)
Other csDMARD, n (%)	6 (2.6%)	2 (2.6%)	4 (2.6%)
**Ongoing b/tsDMARD**	98 (43%)	30 (40%)	68 (45%)
Adalimumab, n (%)	23 (10%)	5 (6.6%)	18 (12%)
Certolizumab pegol, n (%)	16 (7.0%)	8 (11%)	8 (5.3%)
Etanercept, n (%)	31 (14%)	9 (12%)	22 (14%)
Golimumab, n (%)	7 (3.1%)	3 (3.9%)	4 (2.6%)
Infliximab, n (%)	19 (8.3%)	4 (5.3%)	15 (9.9%)
Secukinumab, n (%)	1 (0.4%)	0 (0%)	1 (0.7%)
Apremilast, n (%)	1 (0.4%)	1 (1.3%)	0 (0%)

Mean (SD) if not otherwise stated. ^a^ Based on questionnaire developed by the Swedish National Board of Health and Welfare, with higher values indicating better adherence to Nordic nutrition recommendations. ^b^ Mean of right and left lateral lumbar flexion. ^c^ Previous or current SI joint bone marrow edema according to the ASAS definition. ^d^ ≥1 ICD-10 diagnostic code for any of the following conditions registered in the Skåne Healthcare Register during 10 years prior to the time of examination: gastritis or gastroesophageal reflux disease, peptic ulcer, coeliac disease, microscopic colitis, diverticular disease, malignant neoplasms of the digestive tract. ^e^ One r-axSpA patient had both methotrexate and sulfasalazine ongoing. Missing data for nr-axSpA/r-axSpA, n (%): smoking 0 (0%)/1 (0.7%); dietary index 1 (1.3%)/3 (2.0%); symptom duration 1 (1.3%)/1 (0.7%); back pain ≥ 3 months with onset < 45 years 0 (0%)/3 (2.0%); back pain ≥ 3 months improved by exercise and not relieved by rest 0 (0%)/5 (3.3%); chest expansion 1 (1.3%)/0 (0%); Other gastrointestinal comorbidity 4 (5.3%)/12 (7.9%); ASDAS 2 (2.6%)/6 (3.9%); BASDAI 3 (3.9%)/4 (2.6%); BASFI 3 (3.9%)/5 (3.3%); BASMI 1 (1.3%)/1 (0.7%); VAS pain and VAS global 1 (1.3%)/4 (2.6%); CRP 0 (0%)/1 (0.7%); ASAS 3-month NSAID score 1 (1.3%)/2 (1.3%)

### mSASSS in relation to F-calprotectin

**Fig. 1:**
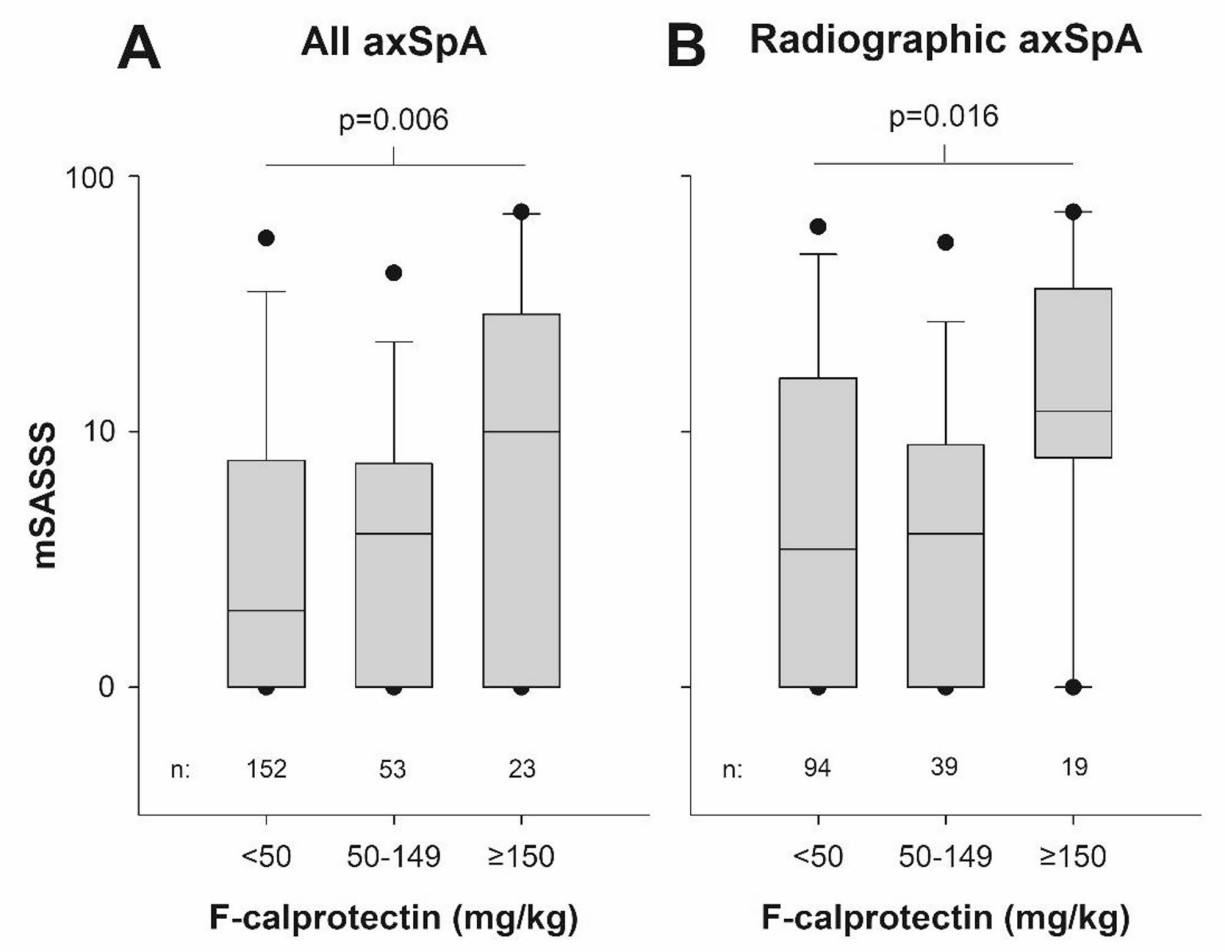
Box plots showing mSASSS distributions stratified for various F-calprotectin levels: **(A)** among all axSpA patients (nr-axSpA + r-axSpA; *n* = 228); **(B)** limited to r-axSpA (*n* = 152). F-calprotectin categorized as normal values (< 50 mg/kg), reflecting no gut inflammation; moderately elevated values 50–149 mg/kg, reflecting some gut inflammation; distinctly elevated values ≥ 150 mg/kg, reflecting evident gut inflammation. Y-axes represent Log_10_-scales. *P*-values for overall between-group comparisons of Log_10_-transformed (due to skewness) mSASSS by one-way ANOVA are displayed above the graphs, and the number of observations in each group are shown below the graphs. Lines represent medians, boxes 25th/75th percentiles, whiskers 10th/90th percentiles and dots 5th/95th percentiles

In the entire axSpA study population, mSASSS differed significantly between patients with various levels of F-calprotectin, with more structural damage in those with higher F-calprotectin (Fig. 1A). Similar results were seen in the analysis restricted to r-axSpA (Fig. 1B).

**Fig. 2:**
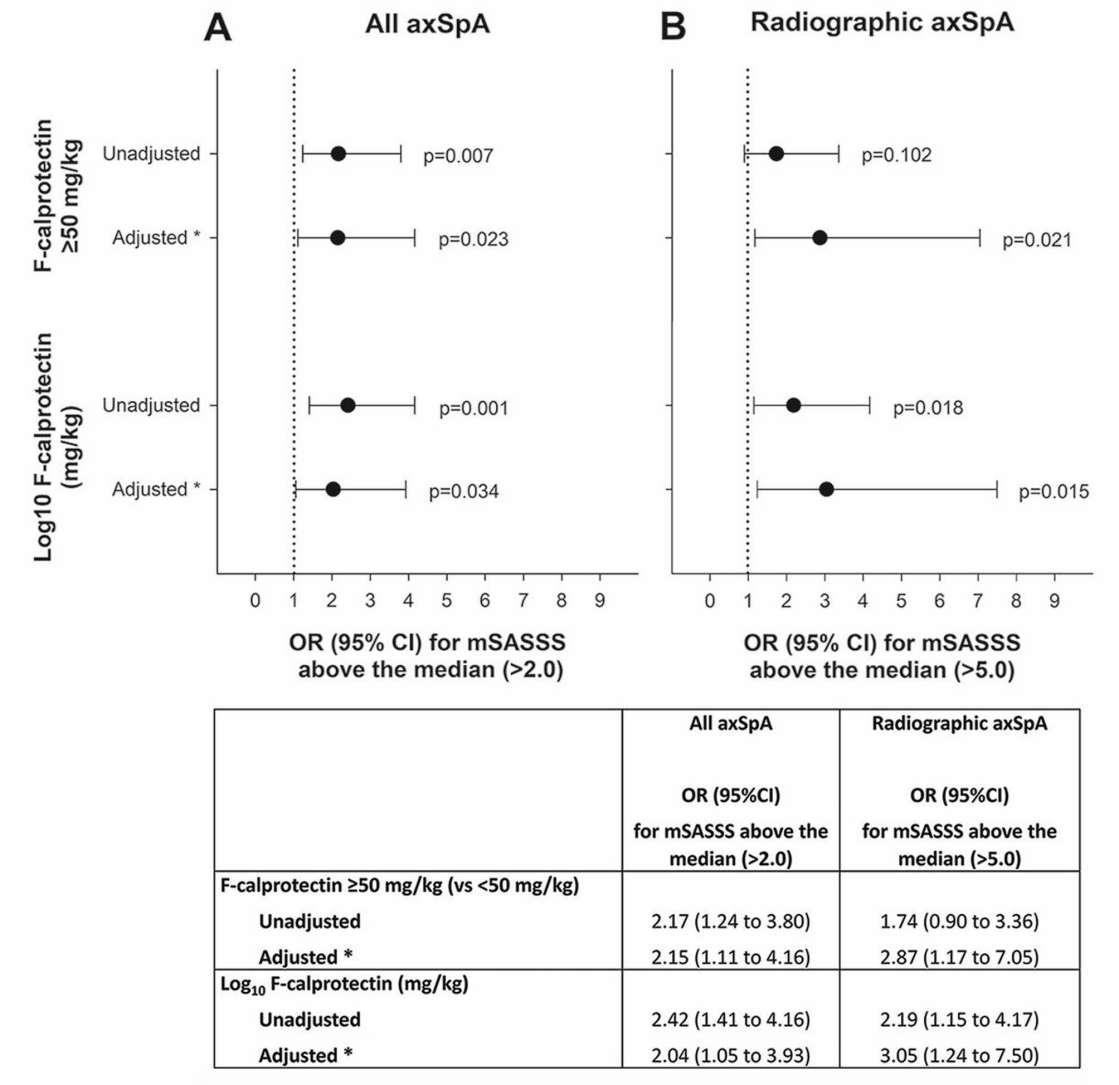
Results of logistic regressions, assessing F-calprotectin in relation to having an mSASSS above the median. Odds ratios (dots) with 95%CI:s (whiskers) for having an mSASSS above the median of those assessed, comparing patients with elevated versus normal F-calprotectin (≥ 50 versus < 50 mg/kg), as well as in relation to higher F-calprotectin, applied as a continuous measure (Log_10_-transformed, due to skewness). **(A)** Results for all axSpA patients (nr-axSpA + r-axSpA; *n* = 228), with a median mSASSS of 2.0. **(B)** Results separately for r-axSpA (*n* = 152), with a median mSASSS of 5.0. Estimates presented both crude and adjusted for known risk factors for structural damage: * sex, symptom duration, HLA-B27 status, smoking (ever/never), CRP (as continuous measure; Log_10_-transformed due to skewness), ASAS 3-month NSAID-score (Log_10_-transformed due to skewness) and anti-TNF therapy use (yes/no), at the time of examination

Among all axSpA patients, both an elevated F-calprotectin ≥ 50 mg/kg and a higher F-calprotectin as a continuous measure were significantly associated with an mSASSS above the median by logistic regression, and this remained also after adjustments (Fig. 2A). When restricted to r-axSpA, similar results were seen, apart from F-calprotectin ≥ 50 mg/kg not being significantly associated with higher mSASSS univariately, although being so after adjustments (Fig. 2B).

### Sensitivity analysis in patients without inflammatory bowel disease

Excluding the 22 patients with IBD (n[nr-axSpA/r-axSpA] = 3/19) did not alter any results for the entire axSpA study population (Additional file 1, Supplementary Figures S1A and S2A). When restricted to r-axSpA (without IBD), however, neither F-calprotectin ≥ 50 mg/kg, nor a higher continuous F-calprotectin value reached statistical significance in relation to having an mSASSS value above the median (*p* = 0.065 and *p* = 0.058, respectively) in the adjusted logistic regression analyses, whereas all other results remained similar to those of the main analyses (Additional file 1, Supplementary Figures S1B and S2B).

### Sensitivity analysis with additional adjustments

Additional adjustment for dietary index and presence of any non-IBD gastrointestinal comorbidity (out of those assessed; see Additional file 1, including Supplementary Table S2, for further details) did not change any of the conclusions from our main analyses (Additional file 1, Supplementary Table S3). Overall, all assessed associations remained similar or were strengthened as compared to the original results, with statistically significant associations now also observed among r-axSpA patients without comorbid IBD (Additional file 1, Supplementary Table S3).

## Discussion

### Main findings

In this study of well-characterized axSpA patients from the population-based SPARTAKUS cohort, enrolled from a defined area of southern Sweden, gut inflammation, measured by elevated F-calprotectin, was cross-sectionally associated with more structural spinal damage, with the highest damage (mSASSS) levels observed in patients with F-calprotectin ≥ 150 mg/kg, reflecting evident gut inflammation. The association also remained when analyzing r-axSpA separately, as well as after adjustments for known structural damage risk factors, including sex, symptom duration, HLA-B27 status, smoking, CRP and treatments.

### Previous research

A pathogenic link between axSpA and IBD has long been suspected, based on strong clinical and genetic associations. IBD is thought to arise through a complex interaction between genetic and environmental risk factors, gut dysbiosis, increased gut permeability and a chronic immune response [19]. Similar processes are present in axSpA too, including gut dysbiosis reminiscent of (yet distinct from) that in IBD [20] and gut inflammation, with around 50–60% of SpA patients displaying Crohn-like, histologic gut mucosal inflammation [1–3], although mostly asymptomatic. According to one central theory, SpA onset is triggered by such gastrointestinal pathology, causing an IL-23/IL-17-pathway driven inflammation spreading to the joints/spine. This causality has not been conclusively proven, however, and the concurrence of gut and joint/spine inflammation may also be due to a shared genetic predisposition.

Regardless of which, mounting evidence points towards an association between gut inflammation and more active axSpA, defined by clinical indices as well as active sacroiliitis on MRI – both of which are in turn known risk factors for structural damage progression [8–11], and thus compatible with our current results. In the GIANT cohort, histologic gut inflammation was significantly associated with higher Bath ankylosing spondylitis disease activity index (BASDAI) [2], while a Brazilian study similarly showed the degree of histologic findings to be positively correlated with ASDAS [12]. From the SPARTAKUS cohort, we have previously shown that axSpA patients with elevated F-calprotectin (≥ 50 mg/kg) have significantly higher ASDAS, even after adjustments for demographics/treatments, whereas such F-calprotectin elevation was not conclusively linked to gastrointestinal symptoms [5, 21]. Other studies have likewise observed positive correlations between F-calprotectin and BASDAI/ASDAS [6, 13]. Furthermore, both histologic gut inflammation and elevated F-calprotectin have been linked to more active SI-joint BME on MRI in axSpA [14, 15], and the latter also in juvenile SpA [22]. Finally, given that male sex is another known risk factor for structural damage [9], it is noteworthy that histologic gut inflammation is more common among male axSpA patients [2].

Apart from these indirect links to known risk factors (worse disease activity/BME/male sex), few prior axSpA studies have addressed gut inflammation in relation to structural damage. In support of our cross-sectional results, however, in a small (*n* = 49) prospective study from the 1990s, all nr-axSpA patients developing radiographic sacroiliitis displayed persistent histologic gut inflammation [23]. Moreover, a previous SPARTAKUS report found F-calprotectin to be higher in r-axSpA than nr-axSpA [5], as also observed by others [3]. Thus, it could be hypothesized that gut inflammation may be a marker, and potentially a driver, of more active disease, which over time leads to the accumulation of structural damage. Prospective studies are, however, needed to investigate a potential causality of the observed association, in particular regarding spinal changes where prior knowledge is most limited. If confirmed (and found valid also in other axSpA populations with various ethnic/environmental backgrounds), the assessment of gut inflammation biomarkers could be of prognostic value in axSpA.

### Strengths and limitations

The population-based setting and detailed patient characterization within the SPARTAKUS cohort are major strengths of the current study, as are the non-restrictive inclusion criteria to this cohort in regard to disease subtype (nr-axSpA/r-axSpA)/severity/comorbidities/treatments. Hence, we believe our results to be fairly generalizable to the wider axSpA population, although at the same time acknowledging that the enrollment from a limited area of southern Sweden may affect generalizability in relation to patients with other genetic and/or environmental backgrounds. The detailed patient characterization allowed us to adjust our analyses for several potential confounders, including CRP and ongoing treatments, where NSAIDs may cause gastrointestinal lesions/inflammation, while monoclonal antibody-type TNF-inhibitors are used to treat IBD. Despite this, residual confounding due to differences in ongoing/prior therapy cannot be ruled out, with symptom onset in our study population spanning from well before until during the era of biologic anti-rheumatic treatments. Furthermore, while additional adjustment for dietary habits and certain non-IBD gastrointestinal comorbidities did not alter our results, F-calprotectin levels may still have been affected by other, unmeasured factors, such as e.g. ongoing gastrointestinal infection (although such potential bias may have been partially mitigated by the adjustment for CRP). In particular for the subgroup analyses restricted to r-axSpA (including the sensitivity analysis of r-axSpA patients without comorbid IBD), the more limited number of subjects assessed may have increased the risk for type 2 error.

Other central limitations are the cross-sectional study design, from which no conclusions can be drawn regarding causality or direction of the association between gut inflammation and structural damage, the single-reader mSASSS scores (although our reliability test showed a good inter-reader agreement) and the lack of spinal MRI data. The latter would have enabled an assessment of whether F-calprotectin elevation is cross-sectionally associated not only with manifest structural spinal damage, but also with more active inflammation in the spine (as previously shown in the SI-joints [15]), in theory preceding such damage development. It would also be of interest to relate F-calprotectin levels to structural spinal lesions as measured by MRI. Finally, the lack of endoscopic examinations is another important limitation of the current study, the inclusion of which would have been required to distinctly ascertain the presence of gut inflammation in the subjects with F-calprotectin elevation. F-calprotectin may also be less sensitive to detect small-bowel than colonic inflammation. Repeated prior SpA studies have, however, confirmed a good agreement between F-calprotectin elevation and presence of both macroscopic and histologic inflammation of the ileum and/or colon on ileocolonoscopy or capsule endoscopy [3, 15, 24, 25].

## Conclusions

In the current study, gut inflammation, as defined by elevated F-calprotectin, was cross-sectionally associated with more structural spinal damage in patients with established axSpA, as well as separately in r-axSpA, even after adjustments for known risk factors for spinal damage development. Future, prospective studies are, however, needed to investigate a potential causality and direction of this observed association, including whether gut inflammation may be a predictor of spinal radiographic progression in axSpA.

## Supplementary Information

Below is the link to the electronic supplementary material.

**Additional File 1**: **Supplementary Table S1.** Characteristics of included and non-included patients. Results of the sensitivity analysis in patients without inflammatory bowel disease, including **Supplementary Figures S1** and **S2**. Sensitivity analysis with additional adjustment for dietary habits and gastrointestinal comorbidity (other than inflammatory bowel disease), including **Supplementary Tables S2** and **S3**. Additional file containing characteristics of included and non-inlcuded patients; results of the sensitivity analysis in patients without inflammatory bowel disease; and description of and results from the sensitivity analysis with additional adjustments, as indicated above.

## Data Availability

The dataset used and analyzed during the current study is available from the corresponding author on reasonable request (and provided the mandatory ethical and legal approvals).

## Abbreviations

ANOVA: Analysis of variance
AS: Ankylosing spondylitis
ASDAS: Axial spondyloarthritis disease activity score using CRP
ASAS: Assessment of SpondyloArthritis international Society
AxSpA: Axial spondyloarthritis
BASDAI: Bath ankylosing spondylitis disease activity index
BASFI: Bath ankylosing spondylitis functional index
BASMI: Bath ankylosing spondylitis metrology index
BME: Bone marrow edema
b/tsDMARD: Biologic or targeted synthetic disease-modifying anti-rheumatic drug
CI: Confidence interval
CRP: C-reactive protein
csDMARD: Conventional synthetic disease-modifying anti-rheumatic drug
Dnr: Diary number
ELISA: Enzyme-linked immunosorbent assay
F: Fecal
HLA: Human leukocyte antigen
IBD: Inflammatory bowel disease
IL: Interleukin
ICD-10: International classification of diseases, 10th edition
ICC: Intraclass correlation coefficient
IQR: Interquartile range
Log_10_: Common logarithm
mNY: Modified New York
MRI: Magnetic resonance imaging
mSASSS: Modified Stoke ankylosing spondylitis spinal score
nr-axSpA: Non-radiographic axial spondyloarthritis
NSAID: Non-steroidal anti-inflammatory drug
OR: Odds ratio
r-axSpA: Radiographic axial spondyloarthritis
SD: Standard deviation
SI: Sacroiliac
SpA: Spondyloarthritis
TNF: Tumor necrosis factor
VAS: Visual analog scale
