# Supplementary material for: Gut inflammation is associated with structural spinal damage in axial spondyloarthritis – results from the observational SPARTAKUS cohort
Source: Arthritis Res Ther. 2025 Oct 21;27:195. doi: 10.1186/s13075-025-03663-z (PMC12539032; doi:10.1186/s13075-025-03663-z)
Supplement: Supplementary file 1 — Additional File 1: Supplementary Table S1. Characteristics of included and non-included patients. Results of the sensitivity analysis in patients without inflammatory bowel disease, including Supplementary Figures S1 and S2. Sensitivity analysis with additional adjustment for dietary habits and gastrointestinal comorbidity (other than inflammatory bowel disease), including Supplementary Tables S2 and S3. Additional file containing characteristics of included and non-inlcuded patients; results of the sensitivity analysis in patients without inflammatory bowel disease; and description of and results from the sensitivity analysis with additional adjustments, as indicated above. [file 13075_2025_3663_MOESM1_ESM.pdf]

ADDITIONAL FILE 1

Gut inflammation is associated with structural spinal damage in axial spondyloarthritis – results from the observational SPARTAKUS cohort

Johan K. Wallman[1,2], Elisabeth Mogard[1,2], Jonas Sagard[1,2], Kristofer Andréasson[1,2], Jan Marsal[3,4], Fatih Inci[5,6], Mats Geijer[5-7], Tor Olofsson[1,2*], Elisabet Lindqvist[1,2*]

[1] Department of Clinical Sciences Lund, Rheumatology, Lund University, Lund, Sweden

[2] Department of Rheumatology, Skåne University Hospital, Region Skåne, Lund, Sweden

[3] Department of Clinical Sciences Lund, Gastroenterology, Lund University, Lund, Sweden

[4] Department of Gastroenterology, Skåne University Hospital, Region Skåne, Lund/Malmö, Sweden

[5] Department of Radiology, Institute of Clinical Sciences, Sahlgrenska Academy, University of Gothenburg, Gothenburg, Sweden

[6] Department of Radiology, Sahlgrenska University Hospital, Region Västra Götaland, Gothenburg, Sweden

[7] Department of Clinical Sciences, Radiology, Lund University, Lund, Sweden

* Authors Tor Olofsson and Elisabet Lindqvist contributed equally to this study.

TABLE OF CONTENTS

Page 2. **Supplementary Table S1.** Characteristics of included and non-included patients

Page 3. Results of the sensitivity analysis in patients without inflammatory bowel disease: **Supplementary Figures S1** and **S2**

Page 5. Sensitivity analysis with additional adjustment for dietary habits and gastrointestinal comorbidity (other than inflammatory bowel disease), including **Supplementary Tables S2** and **S3**

Page 7. References

Supplementary Table S1. Characteristics of included and non-included patients

	All axSpA<br>Included patients<br>n=228	All axSpA<br>Non-included patients<br>n=38
**Male sex**, n (%)	121 (53%)	22 (58%)
**Age**, years	51 (13)	51 (13)
**Smoking ever**, n (%)	81 (36%)	19 (50%)
**Dietary index** [a], 0-12 points	7.2 (2.1)	6.3 (2.5) *
**Family history of SpA**, n (%)	97 (43%)	13 (34%)
**Symptom duration**, years	25 (14)	27 (15)
**HLA-B27 positive**, n (%)	198 (87%)	29 (78%)
**Back pain ≥3 months:**		
With onset <45 years, n (%)	220 (98%)	36 (95%)
Improved by exercise and not relieved by rest, n (%)	169 (76%)	30 (79%)
**Inflammatory back pain (ASAS definition)**, n (%)	196 (86%)	30 (79%)
**Sagittal lumbar flexion (Modified Schober´s test)**, cm	4.3 (2.9)	4.1 (1.5)
**Lateral lumbar flexion** [b], cm	14 (5.2)	14 (6.7)
**Chest expansion**, cm	5.0 (1.3)	4.8 (2.2)
**Non-radiographic axSpA,** n (%)	76 (33%)	10 (26%)
**Sacroiliitis on plain X-ray**, n (%)	152 (67%)	28 (74%)
**SI joint MRI available**, n (%)	124 (54%)	22 (58%)
SI joint bone marrow edema on MRI [c], n (%)	65 (52%)	8 (36%)
**Good response of back pain to NSAIDs**, n (%)	176 (77%)	30 (79%)
**Elevated CRP in the presence of back pain**, n (%)	139 (61%)	24 (63%)
**Peripheral arthritis**, n (%)	118 (52%)	19 (50%)
**Dactylitis**, n (%)	24 (11%)	5 (13%)
**Heel enthesitis**, n (%)	101 (44%)	14 (37%)
**History of uveitis**, n (%)	95 (42%)	17 (45%)
**Skin and/or nail psoriasis**, n (%)	20 (8.8%)	4 (11%)
**Inflammatory bowel disease**, n (%)	22 (9.6%)	2 (5.3%)
**Other gastrointestinal comorbidity** [d], n (%)	36 (17%)	5 (15%)
**ASDAS**	1.8 (0.9)	2.1 (1.1)
**BASDAI**	3.0 (2.2)	3.3 (2.4)
**BASFI**	2.0 (2.1)	2.5 (2.4)
**BASMI**	3.0 (1.6)	3.3 (1.8)
**VAS pain**, mm	30 (25)	40 (31)
**VAS global**, mm	30 (24)	42 (29) *
**CRP**, mg/L	3.4 (4.9)	5.6 (14)
**ASAS 3-month NSAID score**	31 (41)	36 (42)
**Ongoing csDMARD**	48 (21%) [e]	7 (18%)
Methotrexate, n (%)	29 (13%)	6 (16%)
Sulfasalazine, n (%)	14 (6.1%)	1 (2.6%)
Other csDMARD, n (%)	6 (2.6%)	0 (0%)
**Ongoing b/tsDMARD**	98 (43%)	17 (45%)
Adalimumab, n (%)	23 (10%)	3 (7.9%)
Certolizumab pegol, n (%)	16 (7.0%)	5 (13%)
Etanercept, n (%)	31 (14%)	5 (13%)
Golimumab, n (%)	7 (3.1%)	1 (2.6%)
Infliximab, n (%)	19 (8.3%)	2 (5.3%)
Secukinumab, n (%)	1 (0.4%)	1 (2.6%)
Apremilast, n (%)	1 (0.4%)	0 (0%)

Mean (SD) if not otherwise stated. * $p<0.05$ for between-group comparisons by Chi^2-test or Mann-Whitney U-test, as appropriate. [a] Based on questionnaire developed by the Swedish National Board of Health and Welfare, with higher values indicating better adherence to Nordic nutrition recommendations. [b] Mean of right and left lateral lumbar flexion. [c] Previous or current SI joint bone marrow edema according to the ASAS definition. [d] ≥1 ICD-10 diagnostic code for any of the following conditions registered in the Skåne Healthcare Register during 10 years prior to the time of examination: gastritis or gastroesophageal reflux disease, peptic ulcer, coeliac disease, microscopic colitis, diverticular disease, malignant neoplasms of the digestive tract. [e] One included patient had both methotrexate and sulfasalazine ongoing. Missing data for included/non-included patients, n (%): smoking 1 (0.4%)/0 (0%); dietary index 4 (1.8%)/0 (0%); symptom duration 2 (0.9%)/0 (0%); HLA-B27 0 (0%)/1 (2.6%); back pain ≥3 months with onset <45 year 3 (1.3%)/0 (0%); back pain ≥3 months improved by exercise and not relieved by rest 5 (2.2%)/0 (0%); chest expansion 1 (0.4%)/0 (0%); Other gastrointestinal comorbidity 16 (7.0%)/4 (11%); ASDAS 8 (3.5%)/3 (7.9%); BASDAI 7 (3.1%)/5 (13%); BASFI 8 (3.5%)/6 (16%); BASMI 2 (0.9%)/1 (2.6%); VAS pain and VAS global 5 (2.2%)/1 (2.6%); CRP 1 (0.4%)/0 (0%); ASAS 3-month NSAID score 3 (1.3%)/0 (0%). ASDAS, axial spondyloarthritis disease activity score using CRP; ASAS, Assessment of SpondyloArthritis international Society; AxSpA, axial spondyloarthritis; BASDAI, Bath ankylosing spondylitis disease activity index; BASFI, Bath ankylosing spondylitis functional index; BASMI, Bath ankylosing spondylitis metrology index; b/tsDMARD, biologic or targeted synthetic disease-modifying anti-rheumatic drug; CRP, C-reactive protein; csDMARD, conventional synthetic disease-modifying anti-rheumatic drug; HLA, human leukocyte antigen; MRI, magnetic resonance imaging; NSAID, non-steroidal anti-inflammatory drug; SD, standard deviation; SI, sacroiliac; SpA, spondyloarthritis; VAS, visual analog scale.

RESULTS OF THE SENSITIVITY ANALYSIS IN PATIENTS WITHOUT INFLAMMATORY BOWEL DISEASE

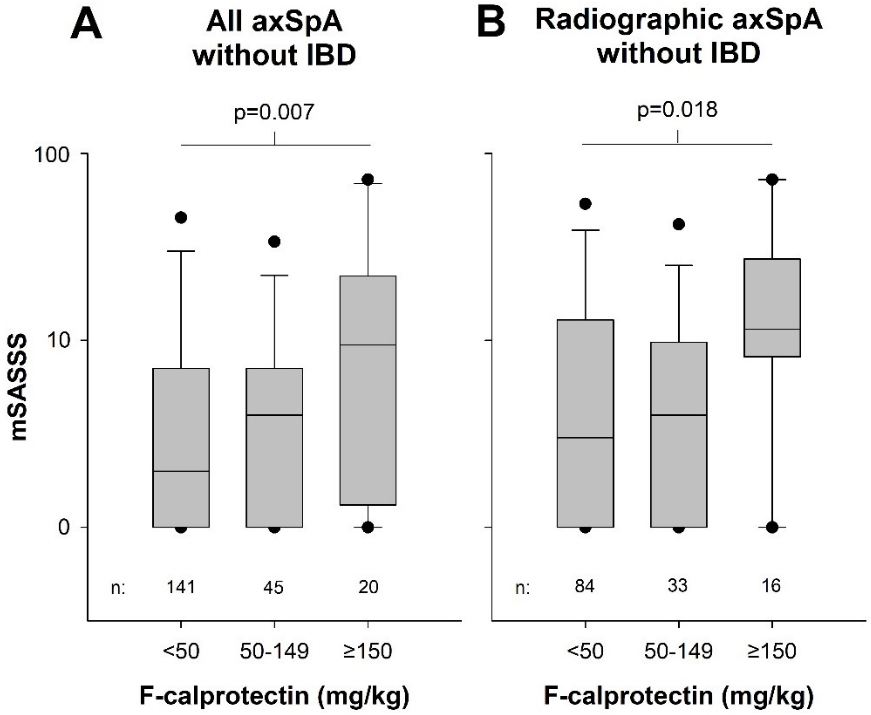

Supplementary Figure S1. Box plots showing mSASSS distributions stratified for various F-calprotectin levels, after exclusion of subjects with comorbid IBD (n=22): **A**. among all axSpA patients (nr-axSpA+r-axSpA; n=206); **B**. limited to r-axSpA (n=133). F-calprotectin categorized as normal values (<50 mg/kg), reflecting no gut inflammation; moderately elevated values 50-149 mg/kg, reflecting some gut inflammation; distinctly elevated values ≥150 mg/kg, reflecting evident gut inflammation. Y-axes represent Log_{10}-scales. P-values for overall between-group comparisons of Log_{10}-transformed (due to skewness) mSASSS by one-way ANOVA are displayed above the graphs, and the number of observations in each group are shown below the graphs. Lines represent medians, boxes 25th/75th percentiles, whiskers 10th/90th percentiles and dots 5th/95th percentiles.

ANOVA, analysis of variance; axSpA, axial spondyloarthritis; F, fecal; IBD, inflammatory bowel disease; mSASSS, modified Stoke ankylosing spondylitis spinal score; nr-axSpA, non-radiographic axSpA; r-axSpA, radiographic axSpA.

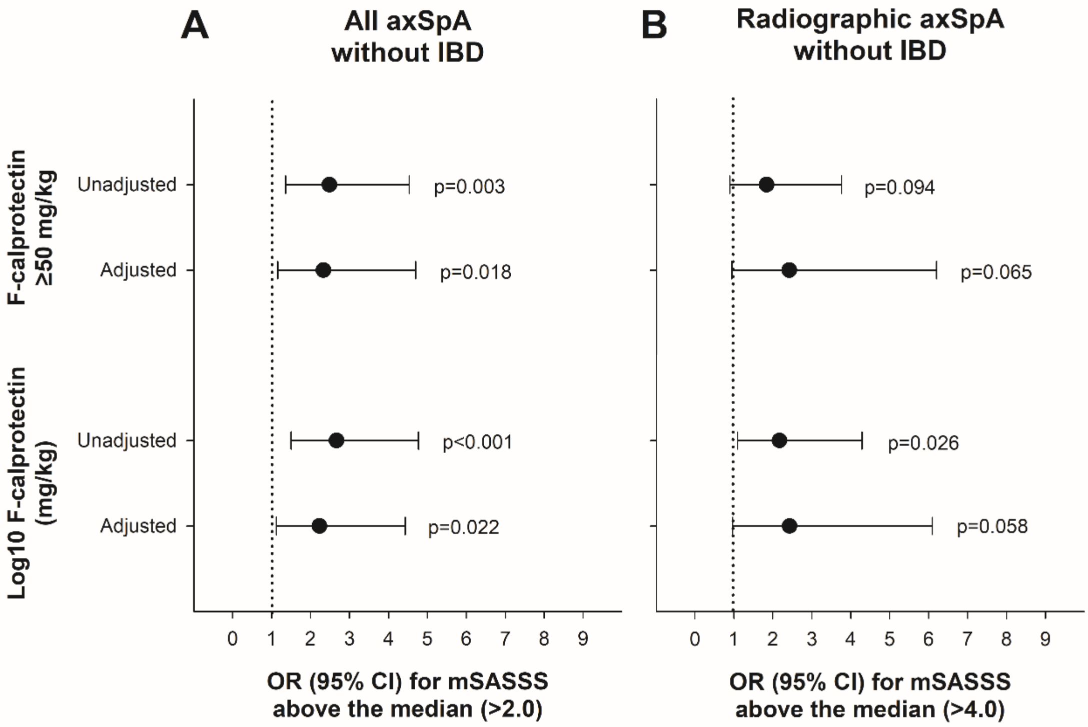

	All axSpA without IBD<br><br>OR (95%CI) for mSASSS above the median (>2.0)	Radiographic axSpA without IBD<br><br>OR (95%CI) for mSASSS above the median (>4.0)
**F-calprotectin ≥50 mg/kg (vs <50 mg/kg)**		
**Unadjusted**	2.48 (1.36 to 4.53)	1.84 (0.90 to 3.76)
**Adjusted ***	2.33 (1.16 to 4.70)	2.42 (0.95 to 6.20)
**Log_{10} F-calprotectin (mg/kg)**		
**Unadjusted**	2.67 (1.50 to 4.77)	2.17 (1.10 to 4.29)
**Adjusted ***	2.23 (1.12 to 4.43)	2.43 (0.97 to 6.09)

Supplementary Figure S2. Results of logistic regressions, assessing F-calprotectin in relation to having an mSASSS above the median, after exclusion of patients with comorbid IBD. Odds ratios (dots) with 95% CI:s (whiskers) for having an mSASSS above the median of those assessed, comparing patients with elevated versus normal F-calprotectin (≥50 versus <50 mg/kg), as well as in relation to higher F-calprotectin, applied as a continuous measure (Log_{10}-transformed, due to skewness). **A.** Results for all axSpA patients (nr-axSpA+r-axSpA) without comorbid IBD (n=206), with a median mSASSS of 2.0. **B.** Results separately for r-axSpA without comorbid IBD (n=133), with a median mSASSS of 4.0. Estimates presented both crude and adjusted for known risk factors for structural damage: * sex, symptom duration, HLA-B27 status, smoking (ever/never), CRP (as continuous measure; Log_{10}-transformed due to skewness), ASAS 3-month NSAID-score (Log_{10}-transformed due to skewness) and anti-TNF therapy use (yes/no), at the time of examination.

ASAS, Assessment of SpondyloArthritis international Society; axSpA, axial spondyloarthritis; CRP, C-reactive protein; F, fecal; HLA, human leukocyte antigen; IBD, inflammatory bowel disease; mSASSS, modified Stoke ankylosing spondylitis spinal score; nr-axSpA, non-radiographic axSpA; NSAID, non-steroidal anti-inflammatory drug; r-axSpA, radiographic axSpA; TNF, tumor necrosis factor.

SENSITIVITY ANALYSIS WITH ADDITIONAL ADJUSTMENT FOR DIETARY HABITS AND GASTROINTESTINAL COMORBIDITY (OTHER THAN INFLAMMATORY BOWEL DISEASE)

At the time of examination, dietary habits were assessed by a questionnaire developed by the Swedish National Board of Health and Welfare [1]. This encompasses four questions regarding the frequencies of intake of a) vegetables and/or root vegetables; b) fruits and/or berries (including juices); c) fish or seafood; d) pastries, chocolate/candy, chips or soft drinks. Each question is scored as 0-3 points, rendering a summarized dietary index of 0-12 points, with higher values indicating an intake more in line with Nordic nutrition recommendations [2] (≤4 points = considerably unhealthy dietary habits; 9-12 points = in general following the Nordic nutrition recommendations) [1].

The presence of non-IBD (inflammatory bowel disease) gastrointestinal comorbidities were assessed by means of international classification of diseases (ICD) codes (Swedish version, 10th edition) retrieved from the Skåne Healthcare Register, which keeps information on all public and the vast majority of private healthcare visits in primary/specialized care in Skåne County since 1998 [3]. A comorbidity was considered present if an individual had received ≥1 ICD-10 code, as main or secondary diagnosis, for the relevant condition during a 10-year period prior to the date of examination. For patients not having resided within Skåne County during their full 10-year assessment period (non-radiographic axial spondyloarthritis [nr-axSpA] n=4; radiographic axial spondyloarthritis [r-axSpA] n=12), this comorbidity data was considered missing. The following diagnoses were selected for assessment, based on being reasonably prevalent in the general population: gastritis or gastroesophageal reflux disease, peptic ulcer, coeliac disease, microscopic colitis, diverticular disease, malignant neoplasms of the digestive tract (for ICD-10 definitions and frequencies in the current study population, see **Supplementary Table S2**). Non-inflammatory, functional diagnoses, such as e.g. irritable bowel syndrome, were not included.

Supplementary Table S2. Definitions and frequencies of assessed non-IBD gastrointestinal comorbidities

Assessed non-IBD gastrointestinal comorbidities	ICD-10 codes	Frequencies in the current study population [a,b]<br><br>nr-axSpA / r-axSpA, n (%)
**Gastritis or gastroesophageal reflux disease**	K20, K21, K22.1, K22.7, K29, R12	4 (5.6%) / 16 (11%)
**Peptic ulcer**	K25-K28, K31-P [c]	0 (0%) / 5 (3.6%)
**Coeliac disease**	K90.0	1 (1.4%) / 5 (3.6%)
**Microscopic colitis**	K52.8	0 (0%) / 2 (1.4%)
**Diverticular disease**	K57	1 (1.4%) / 8 (5.7%)
**Malignant neoplasms of the digestive tract**	C15-C21, C26.0, C26.8, C26.9	0 (0%) / 0 (0%)

[a] Missing data for nr-axSpA/r-axSpA, n (%): 4 (5.3%)/12 (7.9%). [b] Of the assessed nr-axSpA/r-axSpA patients, 6 (8.3%)/30 (21%) had received ICD-10 codes for ≥1 of the conditions. [c] Diagnostic code from the simplified version of ICD-10, optionally used in Swedish primary healthcare, KSH97-P. IBD, inflammatory bowel disease; ICD-10, international classification of diseases, Swedish version, 10th edition; nr-axSpA, non-radiographic axial spondyloarthritis; r-axSpA, radiographic axial spondyloarthritis.

Since dietary habits and non-IBD gastrointestinal comorbidities may potentially affect fecal (F) calprotectin values, we performed a sensitivity analysis where the adjusted logistic regression analyses (the main results of which are presented in **Figure 2**) were repeated, although with the addition of dietary index (0-12 points) and presence of any of the assessed non-IBD gastrointestinal comorbidities (as one combined variable; yes/no) as further adjustment factors. The same procedure was also repeated after exclusion of patients with comorbid IBD.

Compared to our main results, this additional adjustment did not change any of the conclusions (**Supplementary Table S3**). In the assessment of r-axSpA patients without IBD, the associations were even strengthened, now reaching statistical significance (**Supplementary Table S3**).

Supplementary Table S3. Results of logistic regressions, assessing F-calprotectin in relation to having an mSASSS above the median, when including dietary index and presence of any of the assessed non-IBD gastrointestinal comorbidities as additional adjustment factors

	**All axSpA**<br><br>**OR (95%CI) for mSASSS above the median (>2.0)**	**Radiographic axSpA**<br><br>**OR (95%CI) for mSASSS above the median (>5.0)**
**F-calprotectin ≥50 mg/kg (vs <50 mg/kg)**		
Adjusted [a]	2.52 (1.22 to 5.22); p=0.013	3.54 (1.30 to 9.65);p=0.014
**Log_{10} F-calprotectin (mg/kg)**		
Adjusted [a]	2.46 (1.17 to 5.20); p=0.018	3.05 (1.14 to 8.16); p=0.027
	**All axSpA without IBD**<br><br>**OR (95%CI) for mSASSS above the median (>2.0)**	**Radiographic axSpA without IBD**<br><br>**OR (95%CI) for mSASSS above the median (>4.0)**
**F-calprotectin ≥50 mg/kg (vs <50 mg/kg)**		
Adjusted [a]	2.81 (1.29 to 6.13); p=0.010	3.80 (1.21 to 11.93); p=0.022
**Log_{10} F-calprotectin (mg/kg)**		
Adjusted [a]	2.88 (1.30 to 6.40); p=0.009	3.10 (1.03 to 9.32); p=0.044

[a] Adjusted for the same covariates as in the main analyses (i.e. sex, symptom duration, HLA-B27 status, smoking [ever/never], CRP [as continuous measure; Log_{10}-transformed due to skewness], ASAS 3-month NSAID-score [Log_{10}-transformed due to skewness] and anti-TNF therapy use [yes/no], at the time of examination) plus additionally for dietary index at the time of examination and for having received ≥1 ICD-10 diagnostic code for any of the assessed non-IBD gastrointestinal diseases during 10 years prior to examination (yes/no). ASAS, Assessment of SpondyloArthritis international Society; axSpA, axial spondyloarthritis; CI, confidence interval; CRP, C-reactive protein; F, fecal; HLA, human leukocyte antigen; IBD, inflammatory bowel disease; mSASSS, modified Stoke ankylosing spondylitis spinal score; NSAID, non-steroidal anti-inflammatory drug; OR, odds ratio; TNF, tumor necrosis factor; vs, versus.

REFERENCES

1. Sveriges regioner i samverkan, Nationellt system för kunskapsstyrning hälso- och sjukvård, Nationellt programområde för levnadsvanor. Nationellt vårdprogram vid ohälsosamma levnadsvanor – prevention och behandling. https://vardpersonal.1177.se/globalassets/nkk/nationell/media/dokument/kunskapsstod/vardprogram/levnadsvanor.pdf. Accessed 25 August 2025.

2. Nordic council of ministers. Nordic nutrition recommendations 2012. Integrating nutrition and physical activity. https://norden.diva-portal.org/smash/get/diva2:704251/FULLTEXT01.pdf. Accessed 25 August 2025.

3. Löfvendahl S, Schelin MEC, Jöud A. The value of the Skåne Health-care Register: prospectively collected individual-level data for population-based studies. Scand J Public Health. 2020;48:56-63.
